# A reliable and rapid method for molecular detection of malarial parasites using microwave irradiation and loop mediated isothermal amplification

**DOI:** 10.1186/1475-2875-13-454

**Published:** 2014-11-24

**Authors:** Julia R Port, Christian Nguetse, Selorme Adukpo, Thirumalaisamy P Velavan

**Affiliations:** Institute of Tropical Medicine, University of Tübingen, Wilhelmstrasse 27, 72074 Tübingen, Germany; Fondation Congolaise pour la Recherche Medicale, Brazzaville, Republic of Congo

**Keywords:** Malaria, Microwave irradiation, Plasmodium, Isothermal amplification, LAMP, Diagnostics, Point-of-care

## Abstract

**Background:**

Improved living conditions together with appropriate diagnosis can reduce avoidable malarial deaths substantially. Microscopy remains the gold standard in the diagnosis of malaria. However, rapid molecular diagnostic tests (RmDT) are becoming increasingly important and will, most likely, be the diagnostic techniques of choice in the next years.

**Methods:**

In this study, a rapid and reliable nucleic acid extraction procedure from human blood and malarial parasites using microwave irradiation as a promising platform is described. In addition, a tailored loop mediated isothermal amplification (LAMP) methodology that utilizes hydroxynaphthol blue (HNB) and Bst 2.0 DNA polymerases in molecular detection of malarial parasites is described.

**Results:**

Following microwave irradiation for DNA isolation, conventional PCR assays were able to detect up to five malaria parasites/μl. The LAMP methodology described here was capable to detect as low as one *Plasmodium falciparum* parasite/μl after DNA extraction by microwave irradiation. A turnover time of 45 minutes from nucleic acid extraction to final visual read-out was achieved.

**Conclusions:**

The described procedure offers a cheap, simple and fast method of molecular detection of malaria parasites. This test can easily be performed in basic laboratories. The methodology has been validated as a proof of concept and has specifically be developed for use at low-resource settings. Such RmDTs may aid health providers to make timely therapeutic interventions in malaria endemic regions.

## Background

Malaria remains a major public health problem in sub-Saharan Africa. Approximately 3.4 billion people are at risk of malaria worldwide with an incidence of 207 million cases in 2012 and 627,000 reported deaths[1]. Microscopy of blood smears is still considered the gold standard for diagnosing malaria infections. Microscopy, however, is frequently unable to detect low-density infections and it requires skilled expertise[2–4]. Consequently, rapid diagnostic tests (RDT), using blood obtained from finger pricks, are now widely used, especially where quick and easy diagnosis of malaria is needed[2, 3, 5–9]. A major disadvantage of RDTs is that they are relatively expensive and unable to quantify the degree of parasitaemia. In some cases, lack of sufficient sensitivity of RDTs causes, as does microscopy, failure to detect low-density infections[10, 11]. Highest detection rates and specificity can currently be achieved only with polymerase chain reaction (PCR) or real-time PCR (qPCR) assays. These techniques use expensive equipment and reagents as well as functional molecular biology laboratories and appropriate training of laboratory staff. For field applications these conditions are often not fulfilled. In addition, PCR reactions require a rather long time from DNA extraction to visual read-out by electrophoresis or other confirmatory techniques (~5 hours, if performed in a well equipped laboratory) and are as such not appropriate when rapid diagnoses are needed.

In the last 10 years, isothermal amplifications, especially loop-mediated isothermal amplification (LAMP)[12] has revolutionized the field of molecular diagnostics by offering rapid and reliable approaches at highest sensitivity levels. In 2006, a study described a first set of LAMP primers for malaria[13] and LAMP specific primers for four *Plasmodium* species infecting humans were reported in 2007[14], all targeting the 18 s rRNA region of the *Plasmodium* species. Primers were also designed for the genus *Plasmodium* and for *Plasmodium falciparum*, targeting mitochondrial DNA[15]. A commercial kit for malaria LAMP is currently available from Eiken Chemical Co., Ltd., also targeting this genetic region of plasmodia. This LAMP assay has successfully been tested under field conditions for both the18s rRNA[16] and the mtDNA primers[17, 18] and been successful in clinical evaluation of symptomatic and asymptomatic infections[10]. Under field conditions, the easy endpoint visualization of results such as colourimetric change is most advantageous and, thus, desirable. Due to the nature of LAMP reactions, the method offers unique opportunities. LAMP amplification causes production of magnesium pyro-phosphate precipitates, which may be recorded as a rise in turbidity. Such observations are difficult to make for untrained staff and an extra step of visualization would be advantageous. Similar to gel electrophoresis, where LAMP results are visualized by intercalating substances such as SYBR Green^©^ or ethidium-bromide, hydroxynaphtholblue (HNB) can be applied as a potent detection system. As discussed previously[19], SYBR Green^©^ and HNB, usually applied in metal ion titration[20], show higher sensitivity than calcein, which may also be used in LAMP reactions. HNB has been described to be superior to SYBR Green©, as no further equipment is needed for the final read-out and the risk of contamination is minimized as the dye solution is added directly to the reaction mix before amplification[19]. Calcein, a chemical dye quenched with manganese ions may also serve as a fluorescent dye in LAMP reactions. During amplification, the manganese ions are released by pyrophosphate ions generated, resulting in fluorescence. In addition, the free calcein combines with magnesium ions in the LAMP reaction mixture to enhance fluorescent emissions. Compared to SYBR Green^©^ and calcein, HNB is the cheapest dye and successful use of HNB LAMP has recently been demonstrated to be effective in detecting amplified DNA from malaria parasites[21].

The first critical step in standard molecular diagnostics of infectious agents is extraction of DNA. Due to interference with haem, direct DNA amplification from whole blood is difficult to achieve. Therefore, the DNA extraction and purification steps are crucial for subsequent applications. As LAMP is more robust than are standard PCR assays, the extraction step is not that important. Cheap and easy methods have been described using heat denaturation or chemical lysis. In combination with the Eiken LAMP kit, a "boil and spin" method and the "PURE" method, i.e. a simple DNA isolation technique by water bath or thermoblock, are advised for DNA isolation under field applications. An alternative is offered by employing microwave irradiation, which was first described in 1991[22] and 1995 for hepatitis B virus (HBV) DNA from serum[23], and for extraction of *Toxoplasma gondii* derived DNA in 2010[24]. Downstream applications may directly be performed without prior purification measures.

Here, a microwave extraction method was established, along with a tailored LAMP approach for diagnosing malaria. The microwave extraction method was tested for parasite and human genes derived from clinical samples. Sensitivity was tested in repeated serial dilutions and the quality was compared to reference extraction methods using a commercial kit.

## Methods

### Study samples

Whole venous blood samples were collected either into heparinized tubes (5 mls) or by finger prick (5–10 μl) with blood stored on sterile Whatman filter paper at admission to the Albert Schweitzer Hospital, Lambaréné, Gabon, from patients suffering from severe *P. falciparum* infections[25]. Heparinized samples were kept at -80°C until shipment to Germany at -20°C, while blood spots were air-dried and stored in clean, sealed plastic bags at room temperature. Equal volumes of blood were also collected from healthy adult volunteers in Lambaréné.

### Parasite cultures and repeated dilution series

*Plasmodium falciparum* parasite strain 3D7 were kept in culture, synchronized with D-sorbitol in their ring stages. Culture-adapted parasites were then used for extraction procedures and LAMP assays, applying serial dilutions. In brief, following the determination of the parasitaemia by microscopy, the culture was centrifuged to obtain packed cells which contained 10^7^ cells/μl. Fresh whole blood was subsequently used to spike the culture and dilution series were repeatedly made, ranging from calculated 500,000 parasites/μl to 1 parasite/μl and eventual negativity. Equal volumes of these dilution series were further used for genomic DNA extraction by microwave irradiation and, subsequently, for standard PCR and tailored LAMP assays.

### DNA extraction: microwave irradiation

**Figure 1:**
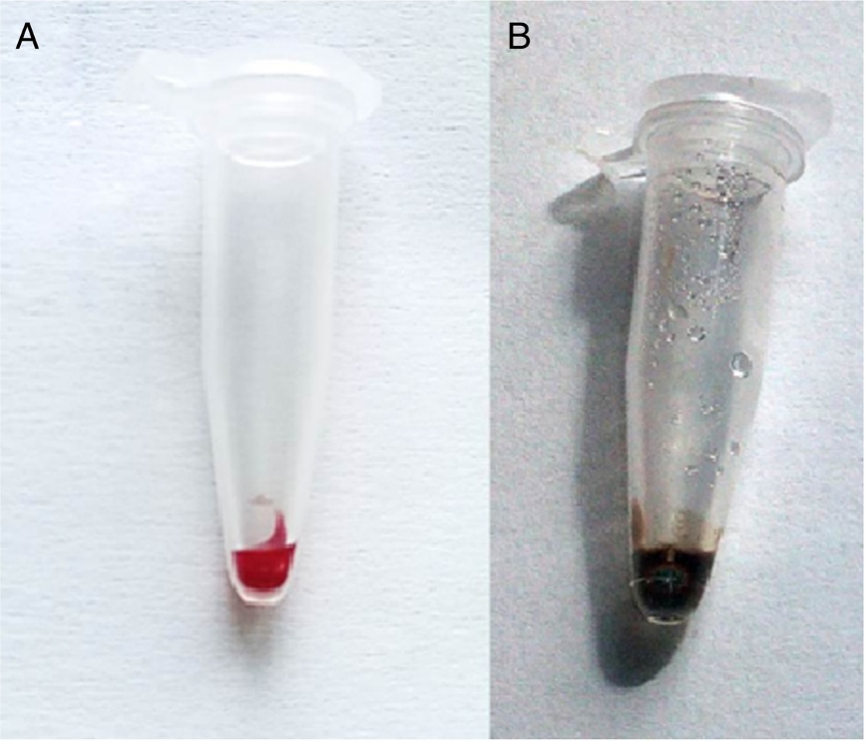
**Microwave irradiated finger prick blood sample 10 μl fresh finger prick blood collected by capillary (A) before and (B) after microwave irradiation.** Formation of vapour and condensed droplets can be observed on the walls and in the lid.

First, DNA was extracted from whole blood samples as well as from the cultured parasites of the dilution series using the conventional QIAamp DNA mini blood kit based extraction procedure (Qiagen, Hilden, Germany) following the manufacturer’s instructions. Next, three standard operation procedures (SOPs) were established for microwave irradiation based DNA extraction (MDA oven, model number: MW17M70G-AU, 230 V, 50 HZ). The three SOPs were designed to apply in processing (i) fresh whole blood, (ii) archived blood samples in heparinized tubes and for (iii) small amounts of blood stored at ambient temperature and eluted from filter paper in 30 μl PBS. Volumes of 10 μl of blood were transferred into 0.5 ml tubes and treated at 800 W for 2 minutes until precipitated and condensed droplets were visible on and retrievable from the tube walls. One μl of the clear precipitated watery solution containing DNA was taken from the walls or the lid of the tube for further use (Figure 1). Alternatively, for enduring storage, 30 μl of sterile phosphate buffer saline (PBS) were added to the irradiated sample. Notably, smaller tubes than those of 0.5 ml volumes should not be used in the irradiation procedure as they might break and be destroyed due to air expansion and, thus, might bear the danger of contamination hazards.

### Amplification using standard PCR

As an example, a nested PCR assay targeting the *pfmdr1* gene of *P. falciparum* was applied as described elsewhere[4]. For each PCR reaction, positive and negative controls were included. Reaction volumes consisted of 20 μl containing 1× Coral load PCR buffer, 0.25 mM of each deoxynucleotide-triphosphate (dNTP), 1 U Taq Polymerase (Qiagen) and 0.2 μM of each primer. The template was 1 μl of the condensed watery solution after DNA extraction by microwave irradiation. Amplification was performed as follows: 35 cycles with an initial denaturation step at 94°C for 5 min, denaturation at 94°C for 30 sec, annealing at 55°C (outer primers) or 60°C (nested primers) for 30 sec, extension at 65°C for 1 min and final extension at 65°C for 5 min. Amplicons were subjected to electrophoresis on a 1.2% agarose gel stained with SYBR green I in 1× Tris-buffered electrophoresis buffer (90 mM Tris-acetate, pH 8.0, 90 mM boric acid, 2.5 mM EDTA). In addition and for confirmation of the specificity of the amplification process, 1 μl of the purified product was directly used as template for direct sequencing, using the BigDye terminator v.1.1 cycle sequencing kit (Applied Biosystems, Foster City, CA, USA) on an ABI 3130XL DNA sequencer. Results were confirmed visually from the sequencing electropherograms. Notably, both the DNA extraction procedure has also be performed to extract human DNA for standard PCR reactions.

### Tailored LAMP assays

#### Primer design and reagents

LAMP primer pairs for *P. falciparum,* as published[14], were provided by Eurofins MWG Synthesis GmbH, Ebersberg, Germany (Table 1). Bst 2.0 WarmStart™ DNA Polymerase (New England BioLabs GmbH, Frankfurt am Main, Germany) was used. This cheap polymerase was specifically designed for high throughput assays and field applications. The polymerase may be stored under up to 45°C without requiring refrigeration. HNB (Fluka, Sigma-Aldrich, St. Luis, USA) was used as the visualization dye. In addition, a 3 mM stock solution of HNB was prepared and stored at room temperature[21, 26]. Isothermal reaction buffer was prepared as a 2× working solution. After thawing, 2.8 mM of dNTPs were added. Betaine solution for enhancing the polymerase activity and reducing the formation of secondary structures in GC-rich regions was purchased from Sigma Aldrich, Munich, Germany.

**Table 1:** **LAMP Primers targeting** ***Plasmodium*** **18 s ribosomal RNA**

Plasmodium spp.	Primers	Sequence 5′ - 3′
**Core primers**	FIP	TCGAACTCTAATTCCCCGTTACCTATCAGCTTTTGATGTTAGGGT
	BIP	CGGAGAGGGAGCCTGAGAAATAGAATTGGGTAATTTACGCG
	F3	GTATCAATCGAGTTTCTGACC
	B3c	CTTGTCACTACCTCTCTTCT
**Loop primers**	LPF	CGTCATAGCCATGTTAGGCC
	LPB	AGCTACCACATCTAAGGAAGGCAG
***P. falciparum***	**Primers**	**Sequence 5′ - 3′**
**Core primers**	FIP	AGCTGGAATTACCGCGGCTG GGTTCCTAGAGAAACAATTGG
	BIP	TGTTGCAGTTAAAACGTTCGTAGCCCAAACCAGTTTAAATGAAAC
	F3	TGTAATTGGAATGATAGGAATTTA
	B3c	GAAAACCTTATTTTGAACAAAGC
**Loop primers**	LPF	GCACCAGACTTGCCCT
	LPB	TTGAATATTAAAGAA

### LAMP Assays

Reactions were performed at 60°C in volumes of 25 μl containing 1.6 μM FIP, 1 μM BIP, 0.2 μM F3, 0.2 μM B3c, 0,2 μM LPB, 0.4 μM LPF, 120 μM HNB, 2× isothermal reaction buffer, 1.4 mM dNTPs, 0.4 mM Betaine solution, 8U Bst polymerase and 1 μL of watery DNA solution as template, following the STARD guidelines for diagnostics. Two heat blocks (Block Thermostat BT200, Kleinfeld Labortechnik, Gehrden, Germany) were used and preheated to 60°C for DNA amplification for to 45 minutes, and enzyme inactivation for 2 minutes at 80°C.

### Visual detection of amplification/endpoint analysis

The amplicon was visualized through a distinct colour change and subsequently confirmed by gel electrophoresis. A change from violet to light sky blue was considered a positive result of amplification. If the reaction remained violet, the sample was assessed as being negative. This assessment was performed at daylight. A positive amplification was determined by a colour change occurring after a minimum of 35 minutes. Absence of colour changes was assigned after 45 minutes. Accordingly, 45 minutes were set as the time needed to achieve a firm result.

## Results

### DNA extraction by microwave irradiation, standard PCR and LAMP assays

Either condensed vapour droplets or blood remains after microwave irradiation diluted in a 30 μl volume of PBS proved to be appropriate templates for further processing in both standard PCR and LAMP assays. DNA extraction was achieved from various sources, including fresh venous blood, heparinized and archived samples and blood eluted from Whatman filter paper. A 0.5 ml tube containing 10 μl of blood inside a microwave oven led to boiling and partial desiccation of the sample and to the formation of vapour, which condenses and may be retrieved from the walls and lids of the tubes (Figure 1). This haem-free condensed vapour contains the nucleic acid.

**Figure 2:**
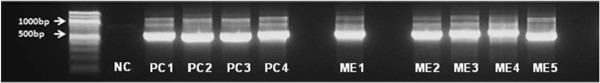
**Amplified** ***pfmdr1*** **gene products after nested PCR.** Standard DNA extraction was carried out using the QIAamp DNA mini blood kit (Qiagen, Hilden, Germany). DNA extraction by microwave irradiation was performed using a microwave oven (MDA, model number: MW17M70G-AU, 230 V, 50 HZ, operated at 800 W). 1 μl of condensed droplets after microwave treatment were utilized for the PCR procedures. First lane: DNA ladder; NC: Negative Control; PC1 and PC2: Standard extraction from archived blood sample and *pfmdr1* amplicons at expected sizes; PC3 and PC4: Standard extraction from 3D7 *P. falciparum* parasites in culture and *pfmdr1* amplicons at expected sizes; ME1 and ME2: Microwave based extraction from archived blood sample and *pfmdr1* amplicons at expected sizes; ME3 and ME4: Microwave based extraction in 3D7 culture parasites and *pfmdr1* amplicons at expected sizes; ME5: Microwave based DNA extraction from fresh blood sample and *pfmdr1* amplicons at expected sizes.

A nested PCR targeting the *pfmdr1* gene of *P. falciparum* was applied and successful amplification of DNA extracted from fresh and archived samples as well as from filter papers by microwave irradiation was achieved (Figure 2). This applied to both, DNA containing vapour droplets and blood remains diluted in PBS. For confirmation of specificity of the amplicon, all PCR products were sequenced and aligned with reference sequences. Correct sequences of amplification targets were confirmed in all cases. The amplicons could successfully be applied to SNP analyses of drug resistance markers. The total time of the entire diagnostic procedure was reduced to less than one hour after sample collection. While maintaining high sensitivity levels, expensive equipment or reagents are not required.

### Analytical sensitivity

**Figure 3:**
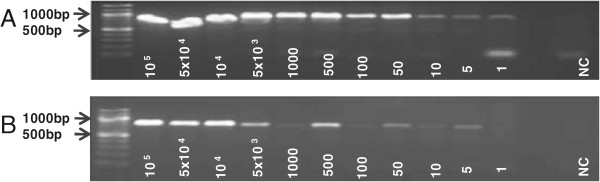
**Limit of detection from standard extraction and microwave irradiation procedure.** Repeated dilution series were made from 100,000 parasites/μl to one parasite/μl until negativity. Equal volumes of diluted culture were further used for DNA extraction and for standard PCR assays. **A**, Serial dilutions including dilutions from 100,000 parasites to negativitiy. The limit of detection was determined to be 1 parasite/μl culture applying the standard extraction procedure (QIAamp DNA mini blood kit); **B**, After microwave based DNA extraction the limit of detection was 5 parasites/μl.

The sensitivity of the PCR assays after microwave-based extraction was established based on repeated dilution series. Heparin blood was spiked with ring stage parasites from 3D7 cultures and serially diluted from 500,000 to one parasite/μl and negativity. DNA was extracted from all samples applying both standard reference methods and microwave irradiation. When using standard DNA isolation methods, one parasite/μl could be reliably detected (Figure 3A). After microwave extraction, five parasites/μl were detected (Figure 3B).

### Loop mediated isothermal amplification

**Figure 4:**
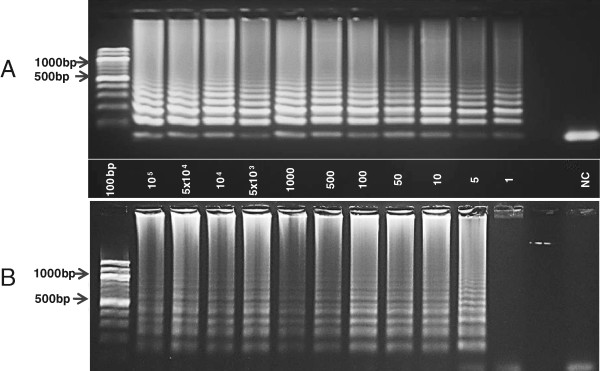
**Limit of detection of parasites extracted by microwave irradiation in tailored LAMP assays.** Successful amplification was characterized by clear colour changes from violet to sky blue and the negative controls remained violet. NC: Negative control; **A**, Pure condensed vapour droplets were used as DNA templates. LAMP can detect 1 parasite/μl culture fluid. **B**, Vapour droplets diluted in PBS. LAMP detects 5 parasites/μl.

**Figure 5:**
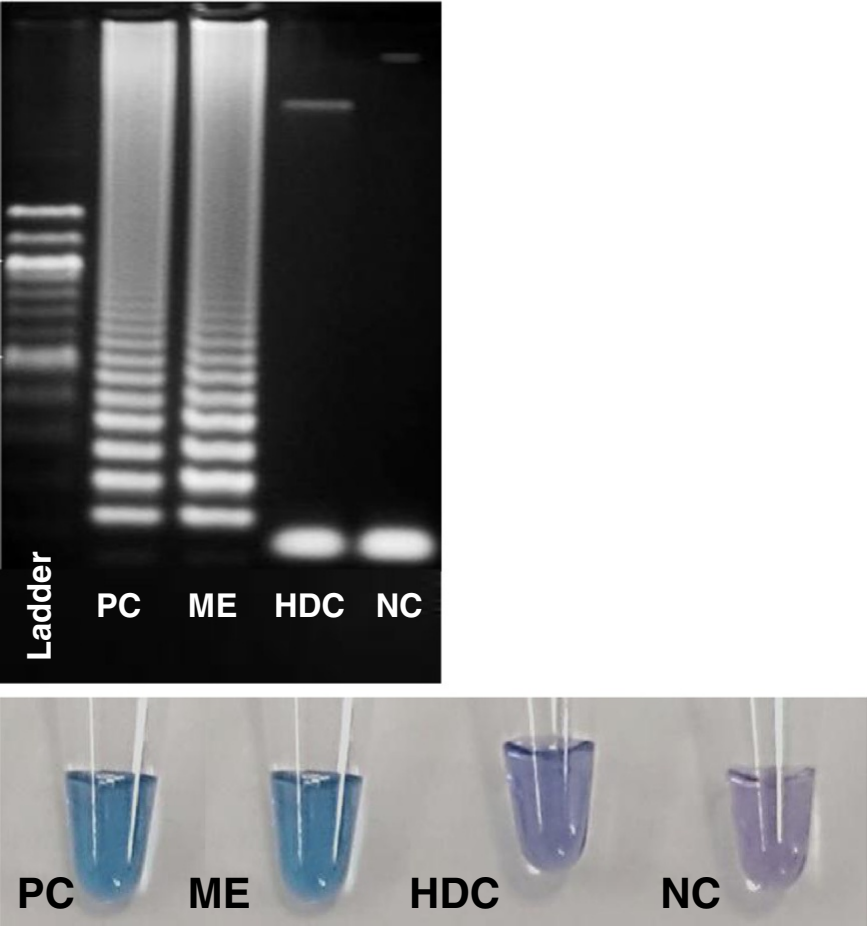
**Visual assessment after 35 minutes following the LAMP assays.** All amplicons were confirmed by gel electrophoresis. PC, positive control after standard DNA extraction; ME, clinical sample, LAMP assay after microwave DNA extraction; HDC, human DNA control; NC, negative control.

No differences in the amplification sensitivity was observed when comparing results obtained by a standard thermocycler or a heating block as proposed here. 3D7 parasites, starting at a dilution of 100,000 parasites/μl to one parasite/μl were subjected to tailored LAMP assays using the DNA extracted by microwave irradiation. The parasite content of the diluted culture was assessed by microscopy, counting 100 oil immersion fields. When direct condensed vapour droplets were used in LAMP, positive amplification was observed with a limit of detection of one parasite/μl (Figure 4A). When blood remains were diluted in PBS, positive amplification was observed at a limit of detection of five parasites/μl (Figure 4B). With HNB used as appropriate dye, negative controls remained violet, while successful LAMP was characterized by a colour change to sky blue. Gel electrophoresis was applied to verify HNB positive results (Figure 5). Repeated tests allowed to set the endpoint of a final decision on test positivity or negativity at a maximum of 45 minutes.

## Discussion

Multiple techniques to extract and purify DNA have been described and applied to date. Problems arising from interference with haem, as occurring in extraction of DNA from venous blood, can easily be circumvented in laboratories when using commercially available extraction kits, while, in low resource settings, commercial DNA extraction kits are not always available. Alternatively, intensive heat treatment or shock freezing can also lyse cells for DNA release. Microwave irradiation has been shown in this study to successfully extract DNA from whole blood samples in less than 3 minutes. No further chemicals are required for isolation or purification. DNA isolated by irradiation could be subjected to both standard PCR amplification and to the LAMP procedure. The use of PBS, although causing a certain decrease of sensitivity due to the dilution, was beneficial, as larger volumes of DNA could be stored. Sensitivity levels observed after microwave irradiation were close to those obtained after extraction with commercial kits.

Thus, microwave irradiation offers a cheap, fast and easy technique that can be applied readily for isothermal amplification and that may be applied in field settings. The nucleic acid extraction by microwave irradiation allows to isolate human DNA and nucleic acid materials from pathogens in blood. Our study demonstrates that DNA may be extracted from fresh blood samples and from archived blood samples, either heparinized or stored on filter papers even after more than a decade. LAMP for malaria has been shown to detect low parasite concentrations[17] and to be successfully applicable in asymptomatic *Plasmodium* infections. When aiming at improving malaria control, simple and easy to perform diagnostic measures detecting subclinical infections need to be applied, as asymptomatic carriers play an crucial role in the transmission cycle of plasmodia and in maintaining endemicity[27–29]. Further and ongoing studies will show, whether the microwave extraction/LAMP system is also appropriate for the detection of low parasitaemia and asymptomatic infections.

An apparent advantage of the system we propose here is the avoidance of contamination through aerosolic PCR-generated DNA fragments, as, due to the HNB dye added to the primary reaction mixture tubes do not need to be opened for further result confirmation. Another value of the technique is that the procedure is easy to perform, does not need technicians trained in molecular techniques and that the final result is obtained by means of visual assessment. This has been performed successfully by untrained personnel after providing them one positive and on negative sample result.

In addition to the obvious advantages, the study has limitations. The minimum size of tubes to be used in microwave exposure should not be below a capacity of 0.5 ml, as through damage of the tube, contamination with biological materials might occur. The problem of potential unavailability of electricity used for irradiation and heating of thermoblocks in the field needs to be addressed by identifying alternative power sources for irradiation and heating. Currently, a prototype that utilizes direct current has been designed for LAMP assays. First trials applying this hand-held battery operated thermoblock for LAMP assays are currently carried out and evaluated.

Taken together, the method described offers a cheap, simple and fast technique of molecular detection of malaria parasites. The method has been validated as a proof of concept to be performed in laboratories with limited resources.
